# *Mitragyna speciosa* Leaf Extract Exhibits Antipsychotic-Like Effect with the Potential to Alleviate Positive and Negative Symptoms of Psychosis in Mice

**DOI:** 10.3389/fphar.2016.00464

**Published:** 2016-12-06

**Authors:** Kamini Vijeepallam, Vijayapandi Pandy, Thubasni Kunasegaran, Dharmani D. Murugan, Murali Naidu

**Affiliations:** ^1^Department of Pharmacology, Faculty of Medicine, University of MalayaKuala Lumpur, Malaysia; ^2^Department of Anatomy, Faculty of Medicine, University of MalayaKuala Lumpur, Malaysia

**Keywords:** apomorphine, bar test, climbing behavior, haloperidol, ketamine, *Mitragyna speciosa*, social withdrawal, vas deferens

## Abstract

In this study, we investigated the antipsychotic-like effect of methanolic extract of *Mitragyna speciosa* leaf (MMS) using *in vivo* and *ex vivo* studies. *In vivo* studies comprised of apomorphine-induced climbing behavior, haloperidol-induced catalepsy, and ketamine-induced social withdrawal tests in mice whereas the *ex vivo* study was conducted utilizing isolated rat vas deferens preparation. Acute oral administration of MMS (50–500 mg/kg) showed an inverted bell-shaped dose-response in apomorphine-induced cage climbing behavior in mice. The effective inhibitory doses of MMS (75 and 100 mg/kg, p.o.) obtained from the apomorphine study was further tested on haloperidol (subcataleptic dose; 0.1 mg/kg, i.p.)-induced catalepsy in the mouse bar test. MMS (75 and 100 mg/kg, p.o.) significantly potentiated the haloperidol-induced catalepsy in mice. Interestingly, MMS at the same effective doses (75 and 100 mg/kg, p.o.) significantly facilitated the social interaction in ketamine-induced social withdrawal mice. Furthermore, MMS inhibited the dopamine-induced contractile response dose-dependently in the isolated rat vas deferens preparations. In conclusion, this investigation provides first evidence that MMS exhibits antipsychotic-like activity with potential to alleviate positive as well as negative symptoms of psychosis in mice. This study also suggests the antidopaminergic activity of MMS that could be responsible for alleviating positive symptoms of psychosis.

## Introduction

Psychosis is a devastating mental illness with a high economic burden for many countries. The molecular mechanisms involved in the pathogenesis of psychosis has been extensively studied and reported in the literature (Saugstad, 2008). In an attempt to reduce the severity of this disease worldwide, many pharmacological treatment options have been constantly developed. Currently available antipsychotics are mainly classified into two categories: typical antipsychotics (e.g., chlorpromazine, haloperidol etc.) and atypical antipsychotics (e.g., clozapine, risperidone etc.). The former class of drugs are effective to treat only the positive symptoms such as delusion, hallucination, and stereotypy behavior by blocking dopamine D_2_ receptors of the mesolimbic pathway in the brain whereas atypical antipsychotics are able to treat both positive as well as negative symptoms such as withdrawal from social contacts, anhedonia, and flattening of emotional responses by blocking D_2_, 5HT_2A,_ and adrenergic (α_2_) receptors (Horacek et al., 2006). Both classes of antipsychotics possess classical adverse effects profile. For example, extrapyramidal motor disturbances such as acute dystonia and tardive dyskinesia are commonly observed with typical antipsychotics whereas weight gain, ventricular arrhythmias, agranulocytosis are some of the common adverse effects of atypical antipsychotics (Howard et al., 2007). Therefore, a search for an alternative antipsychotics to effectively treat both positive and negative symptoms with lesser or no adverse effects is a continuous process in the research world.

Many researchers have opted to natural resources, mostly herbal remedies in order to develop an efficient therapy (Carlini, 2003). *Mitragyna speciosa* Korth. (*M. Speciosa*), commonly known as ketum or kratom, is an ever green tree from coffee family (Rubiaceae) mostly found in Thailand and northern Malaysia. Traditionally, *M. speciosa* leaf has been used to relieve pain, cough, diarrhea, hypertension and it has also been used as a substitute drug to treat opioids addicts (Reanmongkol et al., 2001; Chee et al., 2008). Moreover, *M. speciosa* leaf has been reported for antipyretic, antidiarrheal, analgesic, and local anesthetic activities (Burkill, 1996). Interestingly, *M. speciosa* has been reported for central nervous system stimulant as well as depressant activities at different doses (Chittrakarn et al., 2010). Mitragynine is the main bioactive compound present in the leaf of *M. speciosa* responsible for various CNS activities such as alleviation of ethanol withdrawal symptoms and antidepressant activity in mice (Kumarnsit et al., 2007; Idayu et al., 2011). The pharmacological effects of mitragynine have mainly been explained for its interaction with dopaminergic, adrenergic, and serotonergic receptors (Matsumoto et al., 1996, 1997; Yamamoto et al., 1999; Reanmongkol et al., 2001; Horie et al., 2005; Boyer et al., 2008; Shamima et al., 2012). Moreover, *in vitro* radioligand-binding assays revealed that mitragynine possess inhibitory effect on selected receptors: adrenergic (α_2_) [61.9%], dopamine (D_2_) [54.2%], serotonin [5HT_2C_ (58.8%) and 5HT_7_ (64.4%)] receptors (Boyer et al., 2008). It has also been observed that the long-term consumption of *M. speciosa* leaf darkened the skin although the user remained indoors (Norakanphadung, 1966). The claim for the darker skin of habitual user of *M. speciosa* leaf is intriguing and suggested that it could be due to combination of psychoactive properties and molecular structure of mitragynine (Jansen and Prast, 1988b). It has been reported that the activation of the dopamine type 2 (D_2_) receptors in the rat pituitary gland attenuated the release of α-melanocyte stimulating-like peptides (Kebabian et al., 1984). Therefore, it has been postulated that mitragynine darkened the skin by inhibiting dopamine D_2_ receptors whereby increasing melanocyte-stimulating substances. Jansen and Prast (1988a) proposed if mitragynine proved to be a D_2_ receptor antagonist, it could be effectively utilized to treat psychosis. However, based on our thorough literature search, to date there was no report on antipsychotic activity of *M. speciosa* leaf. Therefore, the present study was aimed to investigate the antipsychotic-like activity of MMS using *in vivo* and *ex vivo* studies.

## Materials and Methods

### Drugs and Chemicals

#### *In vivo* Studies

Methanolic extract of *Mitragyna speciosa* and clozapine (Clozarem^®^, Remedica-Cyprus) solution was prepared as suspension using 1% w/v sodium carboxymethyl cellulose (CMC) solution and administrated orally (p.o.). Haloperidol solution [Manace^®^, Duopharma (M) SDN. BHD, Malaysia] and ketamine hydrochloride solution (Chemistry Department, Ministry of Health, Malaysia) were prepared with normal saline. Apomorphine hydrochloride (Sigma–Aldrich, USA) was dissolved in normal saline containing sodium metabisulphite (0.125% w/v). All drug solutions were prepared fresh and administrated in a constant volume of 1 ml/100g body weight of the animal. The different doses of MMS used in the present *in vivo* study was chosen based on the reported lethal and therapeutic doses of MMS. LD_50_ of MMS was found at 4.90 g/kg in mice (Reanmongkol et al., 2001) and its CNS activities were reported at 50–1000 mg/kg (Reanmongkol et al., 2001; Sabetghadam et al., 2010; Senik et al., 2012).

#### *Ex vivo* Studies

Dopamine hydrochloride and dimethyl sulfoxide (DMSO) (Sigma–Aldrich, USA) were used. Chemicals used to prepare Krebs physiological salt solution were obtained from Sigma–Aldrich, USA. All drug solutions were prepared fresh in double distilled water. The stock solution of different concentrations of MMS was prepared using DMSO (0.1%v/v).

### Plant Collection and Identification

Fresh leaf of *M. speciosa* Korth was collected from Alor Setar Kedah, Malaysia. The leaf was authenticated by Rimba Ilmu, Institute of Biological Sciences, University of Malaya and a voucher specimen (KLU 47980) was deposited for future reference.

#### Preparation of Methanolic Extract of *M. speciosa* (MMS)

Fresh *M. speciosa* leaf (10 kg) was cleaned under running tap water to remove adhering material and dirt, shade dried for 2 weeks and crushed into coarse powder. Two kilograms of the powdered leaf was successively macerated with 10 L of methanol (isocratic HPLC grade, Scharlau, Spain) for 20 h and then proceeded the sonication using water-bath sonicator at room temperature (25°C) for 4 h. The methanol extractive was filtered using a filter paper. Methanol solvent from the extractive was removed by evaporation using rotary evaporator. Approximately, 230 g of dried crude methanolic extract of *M. speciosa* (MMS) was obtained and it was then transferred into an amber air tight container and stored at 4°C until further use.

### LC-MS/MS Analysis and Mitragynine Quantification

The liquid chromatography (LC) analysis was performed using a Shimadzu UFLC system, fitted with a PDA detector (diode array detector, DAD), a column heater-cooler, a vacuum degasser, a quaternary pump, and an autosampler. Separation was done using an XBridge C18 column (2.1 mm × 50 mm, 2.5 μm) and maintained at 40°C. The compound elution was carried out using a linear gradient solvent system consisting of solvent A (0.1% formic acid) and solvent (B) (acetonitrile with 0.1% formic acid) as follows: 0–100% B over 7 min, followed by isocratic elution with 100% solvent (B) from 7 to 15 min, then returned to 10% from 13 min at a flow rate of 0.5 ml/min. The compound separation was monitored with both UV detector at 254 nm and a mass spectrometry detector which operated in positive ionization mode with spectra acquired over a mass range of 50–1000 m/z. A concentration of 5 mg/mL MMS and standard (mitragynine obtained from ChromaDex) stock was prepared in methanol and filtered through a 0.22 nylon filter prior the analysis. The standard calibration graph was plotted with peak area against the serially diluted mitragynine. The amount of mitragynine in the MMS extract was then quantified accordingly using the linearity equation of standard curve.

### Animals

Swiss albino male mice (body weight, 25–30 g) and Sprague-Dawley (SD) male rats (body weight, 250–350 g), obtained from the Animal Experimental Unit, University of Malaya were used in this study. The animals were housed in a group of four in individually ventilated cages. They were fed with standard laboratory food pellet and allowed access to water *ad libitum* and maintained under the standard animal laboratory conditions; 12 h light: 12 h dark cycle (lights off at 19.00 h), 45–55% relative humidity and temperature of 22 ± 1°C. The animals were allowed to acclimatize to laboratory conditions for at least 1 week prior to experiments. All experimental procedures used in this study adhered to the guidelines of the National Research Council of the National Academies of the USA (“Guide for the Care and Use of Laboratory Animals”)(Garber et al., 2011) and were assessed and approved by the Institutional Animal Care and Use Committee, University of Malaya [Ethics No.FARMAKO/18/03/2014/PV(R)].

### *In vivo* Studies

#### Effect of MMS on Apomorphine-Induced Climbing Behavior in Mice

The animals were segregated into eight groups (*n* = 8 in each group). Group I served as a vehicle-control group, received 1% w/v CMC solution (1 ml/100 g, p.o.); Group II was given apomorphine (5 mg/kg, i.p.). Group III, IV, V, VI, VII, and VIII animals were administered with MMS at different doses of 50, 75, 100, 125, 250, and 500 mg/kg, p.o., respectively, 60 min prior to apomorphine injection. The naïve mice were first let to acclimatize to the observation cylindrical metal cage (18 cm × 19 cm) consist of horizontal (4.5 cm apart) and vertical (1 cm apart) metal bars (2 mm) with upper lid for 15 min prior to the experiment. After 15 min acclimatization of the animals in the cylindrical metal cage, they received an intraperitoneal injection of apomorphine (5 mg/kg). Immediately after apomorphine injection, the mice were individually placed at the base of corresponding cylindrical cages. The climbing behavior was assessed every 5 min for 30 min and scored using the following rating scale: 4 = four paws on the wall (Climbing), 2 = two paws on the wall of the cage, 0 = four paws on the floor as described in our earlier publication (Pandy et al., 2012). The total score of 6 time points during 30 min study (every 5 min) was calculated and represent as a climbing index. The maximum possible climbing index was 24. Additionally, the duration of climbing by the animal on the wall and lid of metal cages was recorded and represent as a climbing time for 30 min.

#### Effect of MMS on Catalepsy Produced with Subcataleptic Dose of Haloperidol

This test was conducted using a standard bar test as described elsewhere (Sanberg, 1980). Forepaws of naive mice were placed over a horizontal metal bar (diameter: 3 mm), upraised 4.5 cm from the floor and the time entail to remove the forepaws from the bar was recorded as the duration of catalepsy in seconds. The effective doses of MMS (75 and 100 mg/kg, p.o.) obtained from the apomorphine study or 1% w/v CMC (10 ml/kg, p.o.) was administered 30 min prior to a subcataleptic dose of haloperidol (0.1 mg/kg, i.p.) injection. The subcataleptic dose of haloperidol was chosen in order to differentiate potentiation or reversal effect of drugs on haloperidol-induced catalepsy based on earlier published report (Pandi et al., 2007). The cataleptic behavior of each mouse was recorded at 0 and 60 min after haloperidol injection.

#### Effect of MMS on Ketamine-Induced Social Withdrawal in Mice

The social interaction test was carried out using the method previously described elsewhere (Qiao et al., 2001). Briefly, mice were housed in eight animals per cage (familiar group) and acclimatized to a 12 h reversed light cycle (lights on at 20:00 h) for 2 weeks prior to the experiments. Transparent acrylic box 35 (L) × 20 (W) × 18 (H) cm without a lid was used as social interaction apparatus and the experiments were conducted with a red bulb (15 W) placed above the test box. The mice were introduced to a habituation session prior to the test, whereby each mouse was isolated in the test box for 10 min. During test day, mice were allocated in pairs composed of animal matched for body weight and pertaining to unfamiliar groups (different home cages) that received the same drug treatment. The bodyweights of the paired mice were matched within 1–2 g of variance. Group of mice (*N* = 16) were randomly assigned to treatment groups receiving saline, clozapine (1 mg/kg, p.o.) or MMS (50–500 mg/kg, p.o.) 1 h prior to the test session and ketamine (10 mg/kg, i.p.) was administrated 30 min before the behavioral test. The behavior of each of these pairs was recorded on a camera located above the test box for 10 min for later analysis. All the behavioral observation such as the latency to the first contact and the time spent in social interaction (crawling under or over the partner, genital investigation, following, sniffing and grooming the partner) of each pairs were analyzed by the trained observer who was blind to the treatment group. Passive contact such as lying or sitting with bodies in contact was not considered as a part of social interaction. The test box was wiped clean with alcohol between each trial to remove the evidence of the presences of other mice. Additionally, the locomotor activity was measured using the method described elsewhere (Satow et al., 2009). The locomotion of each mice was accessed as the number of lines (marked on the floor of the box) crossed by the mice during the test.

### *Ex vivo* Studies

#### Effect of MMS on Dopamine-Induced Contractility in the Isolated Rat Vas Deferens Preparation

##### Vas Deferens Tissue Preparation

The SD rats were sacrificed by CO_2_ inhalation and a pair of vas deferens were immediately isolated and carefully cleaned from connective tissues and blood vessels. The epididymal portions of vas deferens were cut into the length of 1 cm and were vertically mounted in 10 mL organ chambers containing 5 mL of Krebs physiological salt solution (KPSS in mM: KCI 4.7, KH_2_PO_4_ 1.2, NaCl 119, NAHCO_3_ 25, MgSO_4_.7H2O 1.2, CaCI_2_.2H_2_O 2.5, and glucose 11.7) at 37°C, gassed with 5% CO_2_ and 95% O_2_ to achieve a desired pH of 7.3–7.4. The tissue was stretched to 0.5 g tension and allowed to equilibrate for 60 min before initiation of experimental protocols. The isometric tension (g) was measured using force transducer connected to the PowerLab recording system (AD Instruments, Australia). During this stabilization period, the bath solution was replaced every 15 min. Following equilibration, the contractile responses of the tissue were tested for viability by the addition of 10% KC1 (high K^+^) for 4 min every 10 min until two consecutive equal contractions were obtained.

#### Dopamine-Induced Contractile Response on Isolated Rat Vas Deferens

To plot the dose-response curve (DRC) of dopamine (DA), a non-cumulative doses of DA (0.4–102.4 μg/mL) were tested on the rat vas deferens preparations as described in our earlier publication (Pandy et al., 2014). From the DRC of DA, a submaximal dose of DA (25.6 μg/mL) was chosen for further investigation. The effect of various concentrations of MMS (1–100 μg/mL, 5 min incubation) or DMSO 0.001% (final concentration) on the contraction of the chosen submaximal dose of DA was investigated. The inhibitory effect of MMS on the contraction induced by the submaximal dose of DA was compared with vehicle control (DMSO). The percentage contractile response of the submaximal dose of DA was calculated with respect to high K^+^ contractile response. Furthermore, the nature of antagonism was examined in the presences of DMSO (0.001%v/v), MMS (1, 5, 10, and 20 μg/ml) or a D_2_ receptor antagonist, haloperidol (1.6, 3.2, 6.4, and 12.8 μg/ml) on the log DRCs of DA.

### Statistical Analysis

All experimental results were analyzed using one-way analysis of variance (ANOVA) followed by Dunnett’s multiple comparison tests. As for the *ex vivo* studies, the concentrations indicated in the text or in the figures represent the final tissue-bath concentration of the drug. The responses were recorded as mean ± standard error of mean (SEM) and “*n*” indicates number of rats used for each set of data. The data were analyzed using Graph Pad Prism version 5.0 statistical software. A level of *p* < 0.05 was considered as statistically significant.

## Results

### LC-MS/MS Analysis and Mitragynine Quantification

**FIGURE 1:**
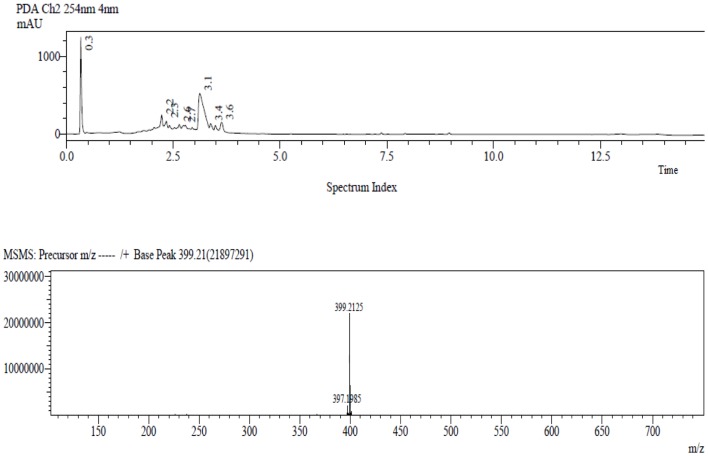
**The LC-MS chromatogram of the target compound, mitragynine, with molecular mass m/z (+) 399.2 was detected at 3.1 min**.

In order to quantify the amount of mitragynine in the MMS extract, a fast and sensitive LC-MS method was developed and used. In **Figure 1**, mitragynine (m/z 399.2) was eluted at 3.1 min. The calibration linearity of mitragynine was reported as *Y* = 0.1811^∗^X + 20.03. The concentration of mitragynine was found to be 4.44 μg /ml of MMS extract, or equally to 4.4% w/w.

### *In vivo* Studies

#### Effect of MMS on Apomorphine-Induced Climbing Behavior in Mice

**FIGURE 2:**
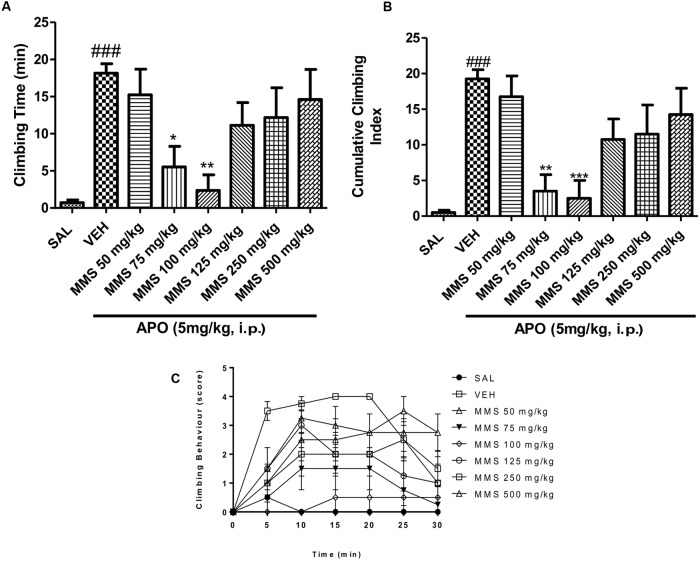
**Effect of methanolic extract of *Mitragyna speciosa* (MMS) (50–500 mg/kg, p.o.) on cage climbing behavior and cage climbing time induced by apomorphine (APO 5 mg/kg, i.p.) in mice. (A)** Total time spent on the wall of the cage. **(B)** The cumulative climbing scores were measured for 30 min after apomorphine administration. **(C)** The climbing behavior was scored at 5 min interval after apomorphine treatment. Each point represents the mean ± SEM from the scores obtained from eight animals. Statistical significance: ^###^*p* < 0.001 compared with the saline control group; ^∗^*p* < 0.05, ^∗∗^*p* < 0.01, ^∗∗∗^*p* < 0.001 compared with the vehicle control (VEH) group.

Methanolic extract of *Mitragyna speciosa* (50–500 mg/kg, p.o.) showed significant inverted bell-shaped inhibitory response on apomorphine-induced climbing time [*F*(7,56) = 4.829; *p* < 0.0003] and climbing behavior [*F*(7,56) = 6.408; *p* < 0.0001]. Dunnett’s *post hoc* analysis revealed that MMS at 75 and 100 mg/kg, p.o., significantly attenuated the apomorphine-induced cage climbing time and climbing behavior as shown in **Figures 2A–C**.

#### Effect of MMS on Catalepsy Produced with Subcataleptic Dose of Haloperidol in Bar Test

**FIGURE 3:**
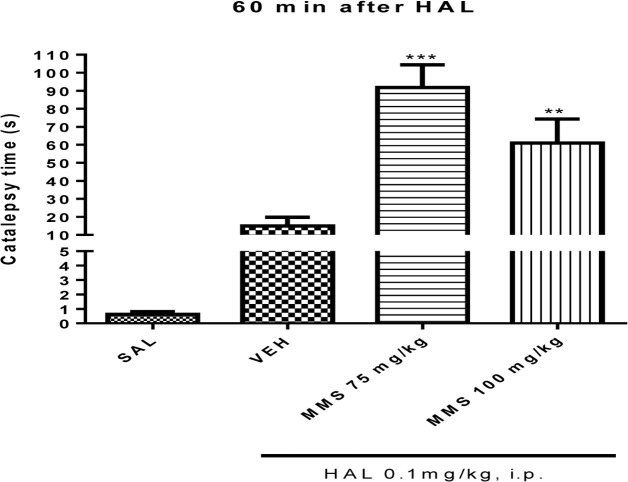
**Effect of MMS (75 and 100 mg/kg, p.o.) on subcataleptic dose of haloperidol (HAL)-induced catalepsy in bar test.** Values are expressed as mean ± SEM. Statistical significance: ^∗∗^*p* < 0.01, ^∗∗∗^*p* < 0.001 compared with the vehicle control (VEH) group; when not indicated the differences were not significant.

The effective doses of MMS (75 and 100 mg/kg, p.o.) obtained from the apomorphine-induced climbing behavior was tested on subcataleptic dose of haloperidol (0.1 mg/kg, i.p.)-induced catalepsy in mice. As shown in **Figure 3**, MMS at doses 75 and 100 mg/kg, p.o., significantly potentiated the haloperidol-induced catalepsy time [*F*(3,28) = 19.51; *p* < 0.0001] in mice.

#### Effect of MMS on Ketamine-Induced Social Withdrawal in Mice

**FIGURE 4:**
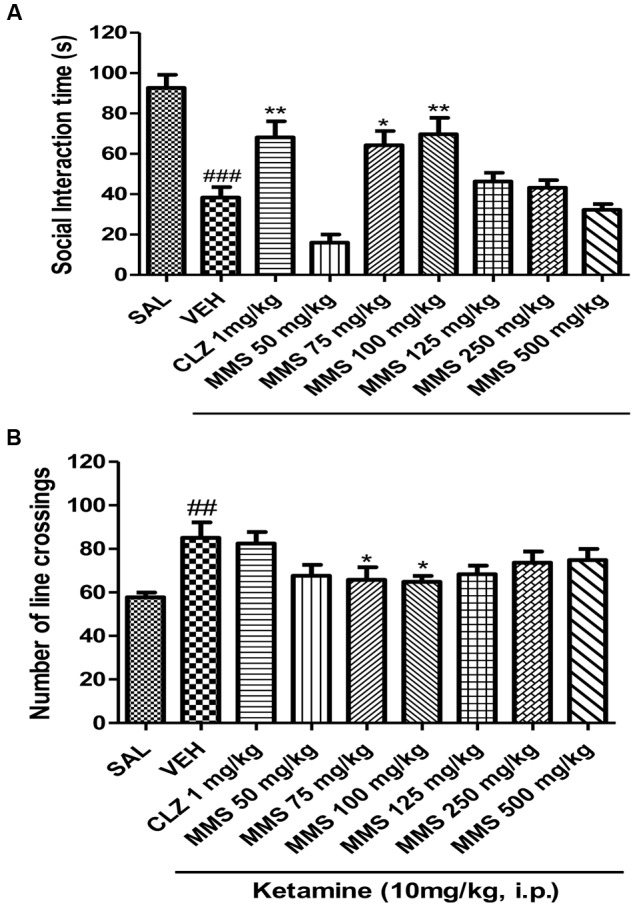
**Effect of MMS (50–500 mg/kg, p.o.) or clozapine (CLZ; 1 mg/kg, p.o.) on ketamine-induced (A)** social interaction time and **(B)** number of line crossings. Statistical significance: ^###^p < 0.001, ^##^*p* < 0.01 compared with the saline control group; ^∗∗^*p* < 0.01, ^∗^*p* < 0.05 compared with the vehicle control (VEH) group.

Ketamine (10 mg/kg, i.p.) significantly induced social withdrawal by attenuating social interaction time between two naive mice when compared with saline treated group (**Figure 4**). MMS (75 and 100 mg/kg, p.o.) significantly reversed the ketamine-induced social withdrawal in mice [*F*(8,63) = 16.17; *p* < 0.0001]. Additionally, it has been observed that ketamine (10 mg/kg, i.p.) significantly (*p* < 0.01) increased the locomotor activity when compared with saline control group. Dunnett’s *post hoc* analysis revealed that MMS at 75 and 100 mg/kg, p.o., also significantly (*p* < 0.05) reversed ketamine-induced hyper motility in mice [*F*(8,135) = 3.149; *p* < 0.0027] as shown in **Figures 4A,B**.

### *Ex vivo* Studies

#### Effect of MMS on Dopamine-Induced Contractility of Isolated Rat Vas Deferens Preparations

**FIGURE 5:**
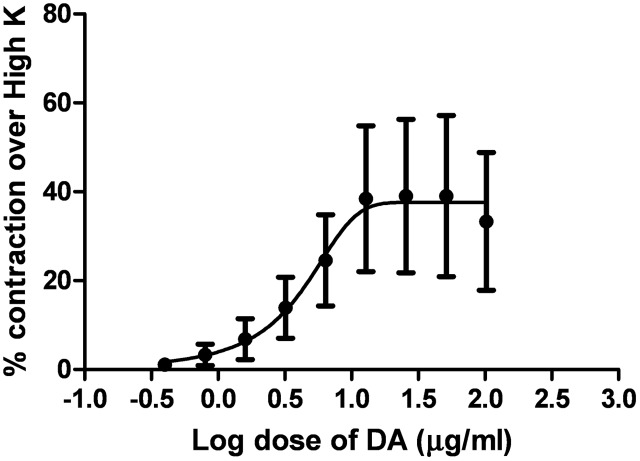
**Log dose response curve of dopamine (DA) expressed as % contraction over 80 mM High K (*n* = 4)**.

**FIGURE 6:**
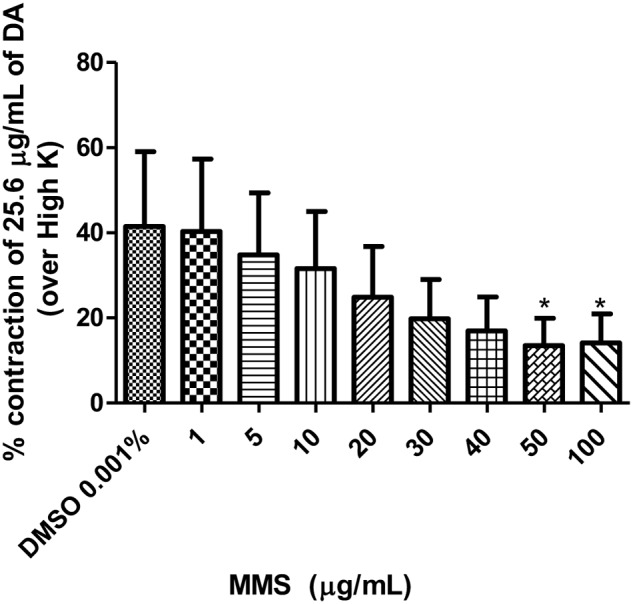
**Effect of MMS (1–100 μg/ml) on DA (25.6 μg/ml)-induced contraction expressed as % contraction over 80 mM High K**. ^∗^*p* < 0.05 compared with DMSO; *n* = 4.

**FIGURE 7:**
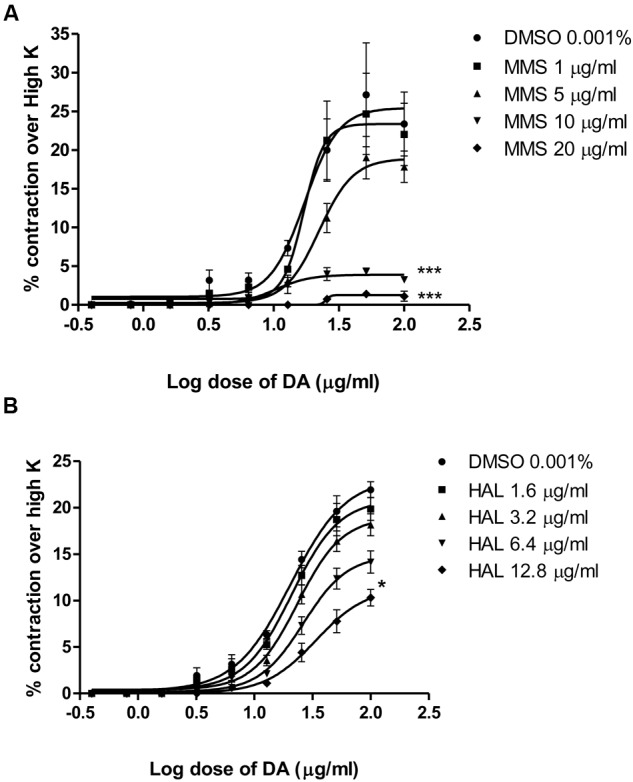
**Log dose response curves of dopamine in the absence and presence of (A)** MMS and **(B)** haloperidol (HAL) expressed as % contraction over 80 mM High K. ^∗^*p* < 0.05, ^∗∗∗^*p* < 0.001 when compared with DMSO; *n* = 4.

Dopamine exhibited dose-dependent contractile response in epididymal segments of the isolated vas deferens preparation (**Figure 5**). A submaximal dose (25.6 μg/mL) of dopamine was chosen from the DRC and it was subsequently used for further studies. MMS at different concentrations (1–100 μg/mL) for 5 min incubation significantly depressed the contractile responses of submaximal dose of dopamine (25.6 μg/mL) in dose-dependent manner as shown in **Figure 6**. In order to evaluate the nature of antagonism by MMS on dopaminergic system, few selected doses of MMS (1, 5, 10, and 20 μg/mL) or haloperidol (1.6, 3.2, 6.4, and 12.8 μg/mL) were tested on DRC of dopamine. MMS and haloperidol, a D_2_ receptor antagonist dose-dependently shifted the DRC of dopamine to rightward with significant reduction in the maximal response (**Figures 7A,B**). However, the pEC_50_ was not significantly altered in both treatment groups ( **Tables 1** and **1**).

**Table 1:** Effect of MMS (1–20 μg/mL) on the pEC_50_ and % maximal contraction (% *E*_max_) of dopamine-induced contractions of isolated rat vas deferens.

Treatment	pEC_50_	% *E*_max_
DMSO (0.001%)	1.23 ± 0.07	27.13 ± 3.66
MMS (1 μg /mL)	1.23 ± 0.07	24.68 ± 3.58
MMS (5 μg /mL)	1.34 ± 0.07	19.02 ± 2.66
MMS (10 μg /mL)	1.01 ± 0.08	4.30 ± 0.60^∗∗∗^
MMS (20 μg /mL)	1.40 ± 0.05	1.40 ± 0.19^∗∗∗^

*^∗∗∗^p < 0.001 when compared with DMSO (0.001%).*

**Table 2:** Effect of haloperidol (1.6–12.8 μg/mL) on the pEC_50_ and % maximal contraction (% *E*_max_) of dopamine-induced contractions of isolated rat vas deferens.

Treatment	pEC_50_	% *E*_max_
DMSO (0.001%)	1.31 ± 0.04	21.94 ± 2.93
HAL (1.6 μg/mL)	1.32 ± 0.04	19.87 ± 2.71
HAL (3.2 (μg/mL)	1.37 ± 0.03	18.17 ± 2.43
HAL (6.4 μg/mL)	1.42 ± 0.04	14.15 ± 1.88
HAL (12.8 μg/mL)	1.53 ± 0.07	10.32 ± 1.30^∗^

*^∗^p < 0.05 when compared with DMSO (0.001%).*

## Discussion

Psychosis animal models are developed based on the neurochemical theory of schizophrenia, primarily involving neurotransmitters such as dopamine and glutamate and it has been used to screen new chemical entities (NCEs) for potential antipsychotic-like effect (Lipska and Weinberger, 2000). Apomorphine-induced cage climbing behavior in mice is a widely used animal model to evaluate the antipsychotic potential of NCEs (Szechtman, 1986). Apomorphine is known to activate both D_1_ and D_2_ dopamine receptors to exhibit these animal behavior (Seeman, 1980; Stoof and Kebabian, 1984). Most of the antipsychotic drugs available in the market are known to suppress the cage climbing behavior by inhibiting the dopaminergic D_2_ receptors (Szechtman, 1986; Davis et al., 1991; Baldessarini and Tarazi, 2001). This study demonstrated that the acute oral treatment of MMS (50, 75, 100, 125, 250, and 500 mg/kg) showed an inverted bell-shaped dose-response relationship in the mouse cage climbing behavior. MMS at doses (75 and 100 mg/kg) significantly decreased the climbing behavior and climbing time in mice. The inhibitory effect of MMS against apomorphine-induced cage climbing behavior suggests the antipsychotic-like effect of MMS mediated by its interaction with the dopaminergic system.

Nucleus accumbens and corpus striatum are commonly suggested as major brain regions associated with antipsychotics-induced catalepsy, which seems as a result of blockade associated with dopaminergic neurotransmission. Besides, a drug which increases the dopaminergic neurotransmission suppresses the neuroleptic-induced catalepsy (Rang et al., 2003). A non-selective dopamine D_2_ blocker, for example, haloperidol induced catalepsy by inhibiting the dopamine D_2_ receptors in the striatum. The ability of the test compounds to potentiate or inhibit the haloperidol-induced catalepsy in rodents is an indicative of a reduction or augmentation of dopaminergic neurotransmission, respectively. In this study, MMS (75 and 100 mg/kg) significantly potentiated catalepsy time induced by subcateleptic dose of haloperidol in mice which implies the antidopaminergic activity of MMS. Interestingly, all tested doses of MMS (50–500 mg/kg, p.o.) *per se* did not affect the mice behavior in bar test (Data not shown). These results suggest that the effective doses of MMS *per se* possessed antipsychotic-like activity without causing extrapyramidal symptoms like catalepsy. Importantly, the antagonism of dopamine D_2_ receptors by the most clinically proven antipsychotic drugs often associated with alleviation of positive symptoms of psychosis such as stereotyped behavior, hallucinations and delusions etc (Gardner et al., 2005). Therefore, it is postulated that MMS could alleviate positive symptoms of psychosis without causing extrapyramidal side effects like catatonia.

To further demonstrate the potential of MMS as an antipsychotic drug for the negative symptoms of psychosis, MMS was tested on ketamine-induced social withdrawal in mice. Ketamine-induced social withdrawal in mice is an established animal model mimicking the negative symptoms of schizophrenic patients (Ellenbroek and Cools, 2000). The antagonism of NMDA receptors by ketamine on the GABAergic neurons leads to decrease the release of GABA, results in the excessive striatal glutamate release, which stimulates the excess release of dopamine, and 5-HT and this causes hyperactivation of D_2_ and 5-HT_2_ receptors, respectively, produce symptoms of hyperactivity (positive symptom) and social interaction deficits (negative symptom) (Homayoun and Moghaddam, 2007; Chatterjee et al., 2012; Uribe et al., 2013). Impressively, in this study, MMS (50, 75, 100, 125, 250, and 500 mg/kg, p.o.) showed a bell-shaped reversal response on ketamine-induced social withdrawal in mice. Moreover, MMS at doses 75 and 100 mg/kg significantly reversed the social deficit induced by ketamine. According to (Corbett et al., 1993), typical antipsychotic drugs deficit the social interaction behavior in rodents whereas atypical antipsychotic drugs tend to facilitate the social interaction behavior. In the present study, a well-known atypical antipsychotic drug, clozapine (1 mg/kg, p.o.) significantly increased the social interaction time in the ketamine-induced social withdrawal animals. This finding is in agreement with previously reported data (Satow et al., 2009) in which acute treatment of clozapine was found to elevate the social interaction time in ketamine-induced social withdrawal mice. However, haloperidol, a typical antipsychotic drug was unable to reverse NMDA receptor agonists (PCP, and MK801)-induced social withdrawal in mice (Qiao et al., 2001; De Moura Linck et al., 2008). One of the main mechanisms involved to alleviate the negative symptoms of psychosis are mediated through inhibition of 5HT_2A_ receptors (Cepeda and Levine, 2006; Stone et al., 2007). Interestingly, mitragynine the main bioactive constituent of *M. speciosa* has been reported to possess the 5HT_2A_ antagonistic-like effect in 5-Methoxy-*N*, *N*-dimethyltryptamine-induced head-twitch response in mice (Matsumoto et al., 1997). Therefore the reversal of ketamine-induced hyperactivity and social withdrawal defects observed with MMS might be mediated through the inhibition of dopamine D_2_ receptors and serotonin 5HT_2A_ receptors.

To further elucidate the dopaminergic mediated actions of MMS, *ex vivo* studies using isolated rat vas deferens preparations were carried out. Dopamine D_2_ receptors are predominantly existed in rat vas deferens and dopamine produce contractile response in rat vas deferens (Boadle-Biber and Roth, 1975). From the data obtained, MMS (1–100 μg/ml) dose-dependently inhibited the contractility evoked by a submaximal concentration of dopamine (25.6 μg/mL). Moreover, the log-dose response curve of dopamine was dose-dependently shifted rightward with a depression of maxima in presence of MMS (1–20 μg/ml) as shown in **Figure 7A** and **Table 1**. The reference drug, haloperidol also showed similar rightward shift with depression of maxima (**Figure 7B**; **Table 2**). However, pEC50 values were not significantly altered at different doses of both MMS (1–20 μg/ml) and haloperidol (1.6–12.8 μg/ml) (**Tables 2** and **2**). These results demonstrate the non-competitive blocking effect of MMS on dopaminergic receptors. To demonstrate the potency of MMS with respect to haloperidol, pEC50 values were compared with haloperidol. pEC_50_ for the MMS (5 μg/mL) is 1.34 ± 0.07 while for haloperidol (6.4 μg/mL) is 1.42 ± 0.04, which indicates that MMS has a similar potency as haloperidol, a dopamine D_2_ blocker. These results confirm the antidopaminergic effect of MMS which is responsible for the antipsychotic-like effect of MMS observed in *in vivo* studies.

Dopaminergic (D_2_)-receptors are the main pharmacological target for treatment of schizophrenia (Missale et al., 1998; Beaulieu and Gainetdinov, 2011). It has been proposed that the positive symptoms are mediated through hyper-activation of mesolimbic dopaminergic D_2_ receptors and the negative symptoms by hypo-activation of mesocortical dopaminergic D_1_ receptors (Toda and Abi-Dargham, 2007). Similarly, glutamatergic NMDA receptors blockade produces both positive and negative psychotic symptoms. For example, dizocilpine, ketamine, and phencyclidine (NMDA antagonists) produce both positive and negative psychotic symptoms in humans (Millan, 2005). Negative psychotic symptoms are also improved by the blockade of 5HT_2A_ receptors. Activation of serotonergic 5-HT_2A_ receptors presents in presynaptic nerve terminals of dopaminergic and glutamatergic neurons inhibit the release of dopamine and glutamate, respectively. Atypical antipsychotics such as clozapine, olanzapine, and risperidone block 5HT_2A_ receptors thereby enhancing the release of dopamine and glutamate in mesocortical circuit and improve the negative symptoms of schizophrenia (Rang et al., 2016). Moreover, clozapine and olanzapine also act as 5-HT_2C_ inverse agonists. 5-HT_2C_ receptor stimulation can also inhibit cortical dopamine release (Katzung and Trevor, 2015). Mitragynine has been shown to inhibit 5-HT_2C_ receptors in *in vitro* radioligand binding assay (Boyer et al., 2008). In present study, MMS at 75 and 100 mg/kg significantly alleviated both positive and negative psychotic-like symptoms in mice. The present results do not clarify the exact neuronal mechanism involved in the antipsychotic-like effect of MMS at 75 and 100 mg/kg. However, it has been postulated that MMS (75 and 100 mg/kg, p.o.) could block dopamine D_2_ receptors and serotonin 5-HT_2A_/5-HT_2C_ receptors thereby improve both positive and negative symptoms of psychosis in mice.

Methanolic extract of *Mitragyna speciosa* at higher doses (>125 mg/kg) did not alleviate both positive and negative symptoms of psychosis in mice. The clinical efficacy of all approved typical and atypical antipsychotic drugs are mainly mediated by blocking post-synaptic D_2_ receptors in the brain (Seeman, 2010). Interestingly, D_2_ receptors also act as autoreceptors in the presynaptic nerve terminals of the dopaminergic neurons and inhibit the synthesis and release of dopamine (Bello et al., 2011; Anzalone et al., 2012). Thus, there is a possibility that MMS at higher doses (125–500 mg/kg) could block pre-synaptic D_2_ receptors thereby stimulate the release of dopamine and lose its antipsychotic effect. Further studies in this direction are warranted to confirm the actual mechanism of action of MMS.

The phytochemical analysis results revealed the presence of mitragynine in MMS by its major peak at a retention time, 3.1 min. 4.44 μg /ml or equally to 4.4% w/w of mitragynine was quantified in MMS which was consistent with earlier published reports (Jansen and Prast, 1988a; Ponglux et al., 1994; Chittrakarn et al., 2010). Mitragynine, being the maximum dominant active alkaloid present in *M. speciosa* is responsible for the diverse pharmacological activities of this plant (Azizi et al., 2010). Interestingly, mitragynine significantly inhibited the dopamine (D_2_) and serotonin (5-HT_2_) receptors in a radioligand-binding assay (Boyer et al., 2008). Therefore, it is hypothesized that antipsychotic-like effect of MMS could be mediated by its main bioactive constituent, mitragynine.

In summary, this study results demonstrated that MMS (75 and 100 mg/kg, p.o.) significantly reversed the apomorphine-induced cage climbing behavior (positive symptoms), attenuated ketamine-induced hyperactivity (positive symptoms), improved ketamine-induced social withdrawal deficits (negative symptoms) and potentiated the haloperidol induced catalepsy in mice, respectively. Further, the antidopaminergic effect of MMS was mediated through the non-competitive antagonism of D_2_ receptors in *ex vivo* study using isolated rat vas deferens preparation. Overall, this study highlighted antipsychotic-like effect of MMS that could be mediated through blockade of dopaminergic (D_2_) and serotonergic (5-HT_2A_/5-HT_2C_) receptors.

## Conclusion

Methanolic extract of *Mitragyna speciosa* is found to be effective in alleviating positive as well as negative symptoms of psychosis in mouse models and it could be mainly mediated through inhibition of D_2_ and 5-HT_2_ receptors. Therefore, MMS could be utilized for the development of novel antipsychotic drug to treat both positive and negative symptoms of psychosis. However, further mechanistic studies to demonstrate antipsychotic-like effect of MMS are warranted. The studies in this direction are currently underway in our laboratory.

## Author Contributions

KV performed all behavioral studies, designed the study, accomplished the data analysis and drafted the manuscript. VP conceived, and designed the study and critically revised the manuscript for important intellectual content. TK and DM helped in *ex vivo* studies. MN critically revised the manuscript for important intellectual content. All authors have read and approved the final manuscript.

## Conflict of Interest Statement

The authors declare that the research was conducted in the absence of any commercial or financial relationships that could be construed as a potential conflict of interest.
